# Immunoprotection of recombinant *Eg*.P29 against *Echinococcus granulosus* in sheep

**DOI:** 10.1007/s11259-016-9656-7

**Published:** 2016-04-19

**Authors:** Hao Wang, Zihua Li, Fu Gao, Jiaqing Zhao, Mingxing Zhu, Xin He, Nan Niu, Wei Zhao

**Affiliations:** Department of Pathogenic Biology and Medical Immunology, Ningxia Medical University, Hui Autonomous Region, Yinchuan, Ningxia 750004 China; Key Laboratory of Hydatid Disease, Ningxia Medical University & Ningxia Institute of Medicine, Hui Autonomous Region, Yinchuan, Ningxia 750004 China; Centre of Scientific Technology of Ningxia Medical University, Hui Autonomous Region, Yinchuan, Ningxia 750004 China; Institute of Clinical Laboratory, Ningxia Medical University, Hui Autonomous Region, Yinchuan, Ningxia 750004 China

**Keywords:** r*Eg*.P29, *Echinococcus granulosus*, Immunoprotection, Vaccine

## Abstract

**Objective:**

This study aims to investigate the immunoprotection of recombinant *E*g.P29 (r*Eg*.P29) vaccine and analyze the underlying mechanism in sheep.

**Methods:**

Three groups of male sheep were immunized subcutaneously with r*Eg*.P29 and PBS, Freund’s complete adjuvant as controls, respectively. After prime-boost vaccination, the sheep were challenged with encapsulated *Echinococcus granulosus* eggs. The percentage of protection in sheep was determined 36 weeks after the infection. Humoral immune response was analyzed for specific IgG, IgG1, IgG2, IgM and IgE levels. Moreover, cytokines including interferon (IFN)-γ, interleukin (IL)-2, IL-4,and IL-10 were also evaluated.

**Results:**

Immunization with r*Eg*.P29 induced protective immune responses up to 94.5 %, compared with immunoadjuvant group. The levels of specific IgG, IgG1, IgG2, and IgE as well as IFN-γ, IL-2, and IL-4 significantly increased after two immunizations (*P* < 0.05); however, the levels of IgM and IL-10 did not show difference.

**Conclusion:**

r*Eg*.P29 showed Immunoprotection and induced Th1 and Th2 immune responses; hence, r*Eg*.P29 is a potential vaccine for *E. granulosus* infection.

## Introduction

*Echinococcus granulosus* (*E. granulosus*) is a cestode parasite that causes endemic zoonosis between human and animals, leading to public health problem and economic loss in the developing and developed countries (Mandal and Mandal 2012; Brunetti and Junghanss 2009). Sheep is a crucial intermediate host in the parasite cycle of *E. granulosus* (Atkinson et al. 2013*)*. Carcasses and offal of sheep after home slaughter are always discarded in poor and remote communities and then dogs scavenge these, completing the parasite cycle and putting communities at risk of cystic echinococcosis (Li et al. 2014). Therefore, as mentioned above, it is very important to control the infection in sheep.

Vaccination is the most effective strategy for the prevention of infectious diseases worldwide. In *E. granulosus* infection, some vaccine candidates have proven to be highly protective in mice (Li et al. 2012; Sun et al. 2011; Shi et al. 2009), goats, sheep and bovines (Read et al. 2009; Chow et al. 2008; Dutton et al. 2012; Gauci et al. 2005; Heath et al. 2012a; Lightowlers et al. 1999; Woollard et al. 2000). Representative vaccine EG95 induced immunoprotection up to 95 % to 96 %. However, due to complicated multi-cellular pathogen and host interplay, there is still no vaccine approved for clinical use. In this study, we will sought to evaluate the potential of *Eg*.P29 as a vaccine candidate for sheep.

*Eg*.P29 was first described by Gonzalez et al. as a novel 29 kDa antigen in hydatid cyst fluid (Gonzalez et al. 2000). Our previous study confirmed that r*Eg*.P29 could protect against *E. granulosus* secondary infection in mice (Shi et al. 2009). However, mice are not the intermediate hosts for *E. granulosus* infection in human. Furthermore, secondary infection in mice is very different from natural infection and the result is not convincing for the mimicking challenge. In the present study, we will evaluate the immunoprotection of recombinant *Eg*.P29 (r*Eg*.P29) against an oral challenge infection with *E. granulosus* eggs in sheep.

## Materials and methods

### Animals and parasites

Thirty male sheep, 4–6 months old, were obtained from Lanzhou Veterinary Research Institute, Chinese Academy of Agricultural Sciences. The sheep were first scanned negative by serological test and then randomly allocated into three groups (eight sheep/group): r*Eg*.P29, Freund’s complete adjuvant (FCA), and PBS groups. An additional 2 sheep/group were prepared for accidental death. The sheep were all raised on the same farm. All experiments were approved by the Ethics Committee of Ningxia Medical University. *E. granulosus* eggs were also obtained from the institute. Before the oral challenge, 3000 freshly collected *E. granulosus* eggs were packaged into each capsule.

### Preparation of rEg.P29

The *Eg.P29* gene was obtained from hydatid cysts of patients in General Hospital of Ningxia Medical University (The Chinese strain of the gene was recorded into GenBank: sequence number AF078931. Plasmid *Eg*.P29/pET28a was constructed and expressed in *Escherichia coli* by our lab previously (Shi et al. 2009)). Briefly, the plasmid *Eg*.P29/pET28a was transformed into *E.coli BL21* (DE3) pLysS, and protein expression was induced at 37 °C for 8 h in the presence of 0.4 mM isopropyl-b-D-thiogalactoside (IPTG, Invitrogen). Subsequently, r*Eg*.P29 with 6 × histidines (His) tag in the C terminus was purified by nickel chelate affinity chromatography (Novagen) according to the manufacturer’s instructions. The purified r*Eg*.P29 was identified with 12 % sodium dodecyl sulfate–polyacrylamide gel electrophoresis(SDS-PAGE) and the Western blot method. Briefly, r*Eg*.P29 was resolved using 12 % SDS–PAGE and electrophoretically transferred onto a nitrocellulose membrane. After blocking with 5 % skim milk at room temperature (RT) for 2 h, the membrane was probed with anti-His-tag mouse monoclonal antibody (MAb) (1:200) or pooled sera (1:200) from sheep infected with *E. granulosus* eggs, at RT for 1 h. After washing, the blot was incubated with horseradish peroxidase (HRP)-conjugated goat anti-mouse IgG or rabbit anti-sheep IgG at RT for 1 h before detection with West Pico Chemiluminescent Substrate (Thermo Scientific, Rockford, IL). The protein concentration was determined using Bradford method (Bradford 1976).

### Vaccination and challenge with *E. granulosus* eggs

Sheep in three groups were subcutaneously vaccinated in the neck region with the corresponding treatments on day 1: PBS group, 100 μl of PBS; FCA group, 50 μl of FCA and 50 μl of PBS; and r*Eg*.P29 group, 50 μg of r*Eg*.P29 (1 μg/μl) emulsified with 50 μl of FCA. The second immunization was administered with the same preparation on day 28, except that FCA was replaced by Freund’s incomplete adjuvant. Four weeks after the last vaccination, each sheep was orally challenged with encapsuled freshly 3000 *E. granulosus* eggs. The percentage of protection in sheep was determined according to the Dempster method (Dempster and Harrison 1995). Immunoprotection is calculated as: protection (%) = (1 − average of cysts in the test group/average of cysts in the control group) × 100.

### Detection of specific antibodies

Serum antibody responses were detected by ELISA at 0, 1, 2, 4, 6, 9, 12, 20, 36, and 44 weeks after immunization. 96-well microplates (Sino-American Biotechnology Company, Beijing, China) were coated with 100 μl of r*Eg*.P29 (0.1 μg/μl) per well and incubated overnight in 0.1 M carbonate buffer (pH 9.6) at 4 °C. Serum samples were diluted (1:100) in PBS with 0.05 % Tween-20 (PBST) and incubated at 37 °C for 1 h in duplicate. Bound antibody was detected by HRP-conjugated goat anti-sheep IgG, IgG subclass, IgE, and IgM (Novagen) at1:1000 dilution in PBST. Optical density (OD) values were read at 490 nm (Bio-Rad).

The sheep were scanned negatively using ELISA coated with hydatid cysts crude antigens. Briefly, 96-well microplates were coated with 100 μl of crude soluble antigens(0.1 μg/μl) from hydatid cysts per well, other steps are as well as above mentioned.

### Determination of cytokines

The OD value of serum cytokine was detected through ELISA in accordance with the manufacturer’s instructions (Jinmei Biotech Company, Beijing, China). Serum samples were diluted (1:100) in PBS and tested in duplicate. Diluted serum was added into the 96-well microplates and incubated for 2 h at 37 °C. After washing with PBST, 50 ng of biotin-conjugated antibody was added to each well and then incubated again for 2 h at 37 °C. After washing, peroxidase-labeled streptavidin was added to the wells and incubated for 1.5 h at 37 °C. The wells were then washed and incubated with the substrate for 0.5 h at 37 °C. Finally, the reaction was terminated by adding 50 μl of 2 M sulfuric acid. The OD value was determined at 490 nm using an ELISA reader (Bio-Rad). A standard curve was prepared to calculate cytokine concentration.

### Statistical analysis

All statistical analyses were performed using Prism 5.0 (GraphPad Software Inc., CA, USA). Data were obtained from at least three independent experiments and represented as mean ± standard error. Statistical analysis was conducted using the non-parametric (Mann-Whitney) t-test or Student’s t-test. Results were considered significant at *P* < 0.05.

## Results

### Expression and purification of rEg.P29

**Fig. 1:**
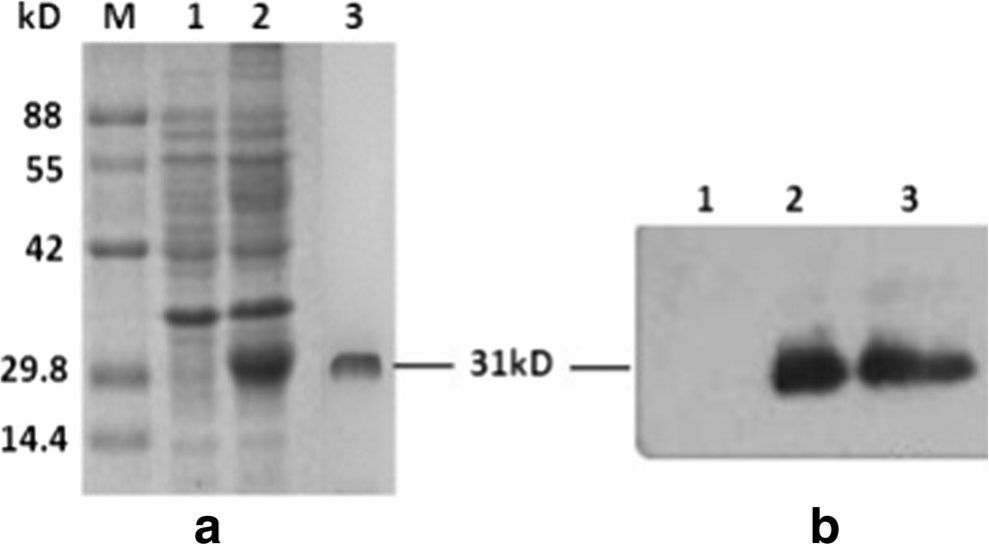
Expression and identification of r*Eg*.P29. Figure 1a shows the results of the SDS-PAGE analysis. The gel was stained by Coomassie brilliant blue: M, molecular weight markers; line 1, *E. coli* lysates without IPTG induction; line2, *E. coli* lysates with IPTG induction; and line 3, purified r*Eg*.P29. Figure 1b shows the results of the Western blot analysis of r*Eg*.P29. Line1, PBS as negative control; line 2, the purified r*Eg*.P29 protein was probed with anti-His-tag mouse MAbs; line 3, the purified r*Eg*.P29 protein was probed with pooled sera from infected sheep

rEg.P29 was successfully expressed and purified. The purity and size of the recombinant protein was identified through SDS–PAGE and the Western blot. The recombinant protein showed a high purity and a molecular weight of 31 kDa as predicted (Fig. 1).

### r*Eg*.P29 immunization reduced the number of developing cysts

**Fig. 2:**
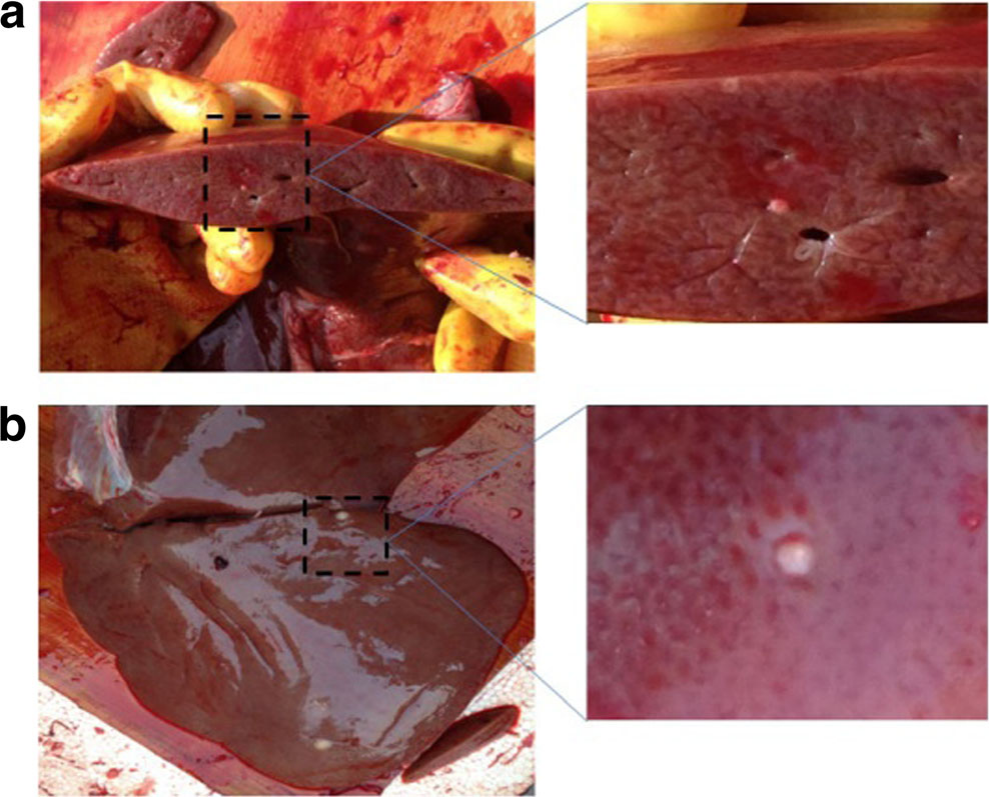
Hydatid cysts in sheep liver. Sheep were euthanized, and the livers were immediately isolated in the laboratory. Cysts on the liver surface were examined carefully; the liver was then sliced into 3 mm pieces to check and record the number of cysts. Fibrotic and calcified cysts were regarded as invalid cysts. Immunoprotection was calculated through cyst reduction method in accordance with the following formula: protection (%) = (1 − average of cysts in the test group/average of cysts in the control group) × 100. Cysts can grow on the surface and inside on the liver. Figure 2a shows cysts inside of the liver. Figure 2b shows cysts on the surface of the liver

The sheep were euthanized at the end of 44 weeks, and cysts were counted to determine the protective effects (Fig. 2). Significant reduction of cyst load was found in the r*Eg*.P29 group compared with the control group which immunized with PBS or FCA at 9 months post-infection (Table 1). The immunoprotection is 94.5 % and 95.1 % (compared with PBS and FCA group).

**Table 1:** The number of hydatid cysts and immunoprotection of the r*Eg*.P29 vaccine in sheep

Group	No. of cysts in individual sheep								Mean ± SEM
PBS	44	34	26	13	22	36	27	20	27.75 ± 3.49
FCA + PBS	41	21	34	19	13	17	30	25	25.00 ± 3.33
r*Eg*.P29	3	1	1	0	2	1	1	2	1.375 ± 0.324*^#^

The number of hydatid cysts in r*Eg*.P29 group shows significant reduced from group PBS (*P < 0.0001) or group FCA + PBS (^#^ *P* < 0.0001) by Student’s t-test.

### r*Eg*.P29 immunization effectively induced humoral responses

**Fig. 3:**
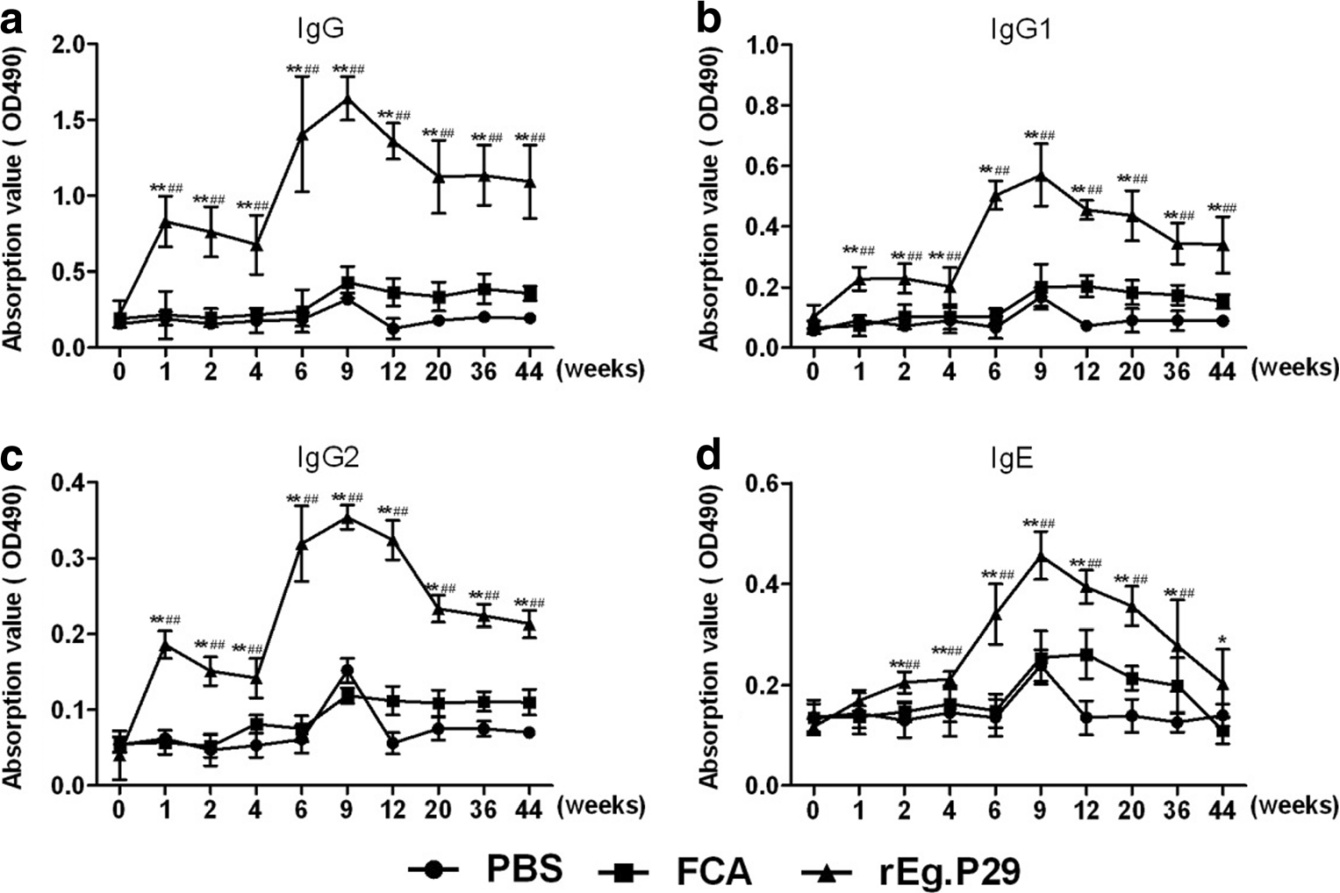
OD value profiles of IgG and IgE in immunized sheep before and after infection. Ten time points were selected for serum antibody examination: week 0 (before immunization), week 1 (first immunization), week 2 (1 week after the first immunization), week 4 (second immunization), week 6 (2 weeks after the second immunization), week 9 (1 week after the infection), week 12 (4 weeks after the infection), week 20 (12 weeks after the infection), week 36 (28 weeks after the infection), and week 44 (36 weeks after the infection). *P < 0.05 and **P < 0.01 indicate significant difference between PBS and r*Eg*.P29 groups. #*P* < 0.05 and ##*P* < 0.01 denote significant difference between FCA and r*Eg*.P29 groups

The immunization with r*Eg*.P29 induced significantly higher levels of specific IgG, IgG1, and IgG2 (Figs. 3a, b, c and d) one week after the first immunization compared with those before the immunization (*P* < 0.01). At 2 and 4 weeks after the first immunization, the levels of IgG, IgG1, and IgG2 reduced slowly but remained statistically different from those in PBS or FCA group (*P* < 0.01). Two weeks after the last immunization, the levels of specific IgG, IgG1, and IgG2 in the rEg.P29 group were higher than those in PBS or FCA group (*P* < 0.01). Specific IgE level was not significantly different until the second week after prime immunization. Interestingly, IgE level slowly increased in the r*Eg*.P29 group and was significantly different (*P* < 0.01) from that in the two control groups respectively. After the second immunization, the levels of specific IgG, IgG1, IgG2, and IgE in the r*Eg*.P29 group significantly increased and peaked at 9 weeks (1 week after the infection).

### r*Eg*.P29 immunization induced Th1/Th2 cytokine responses

**Fig. 4:**
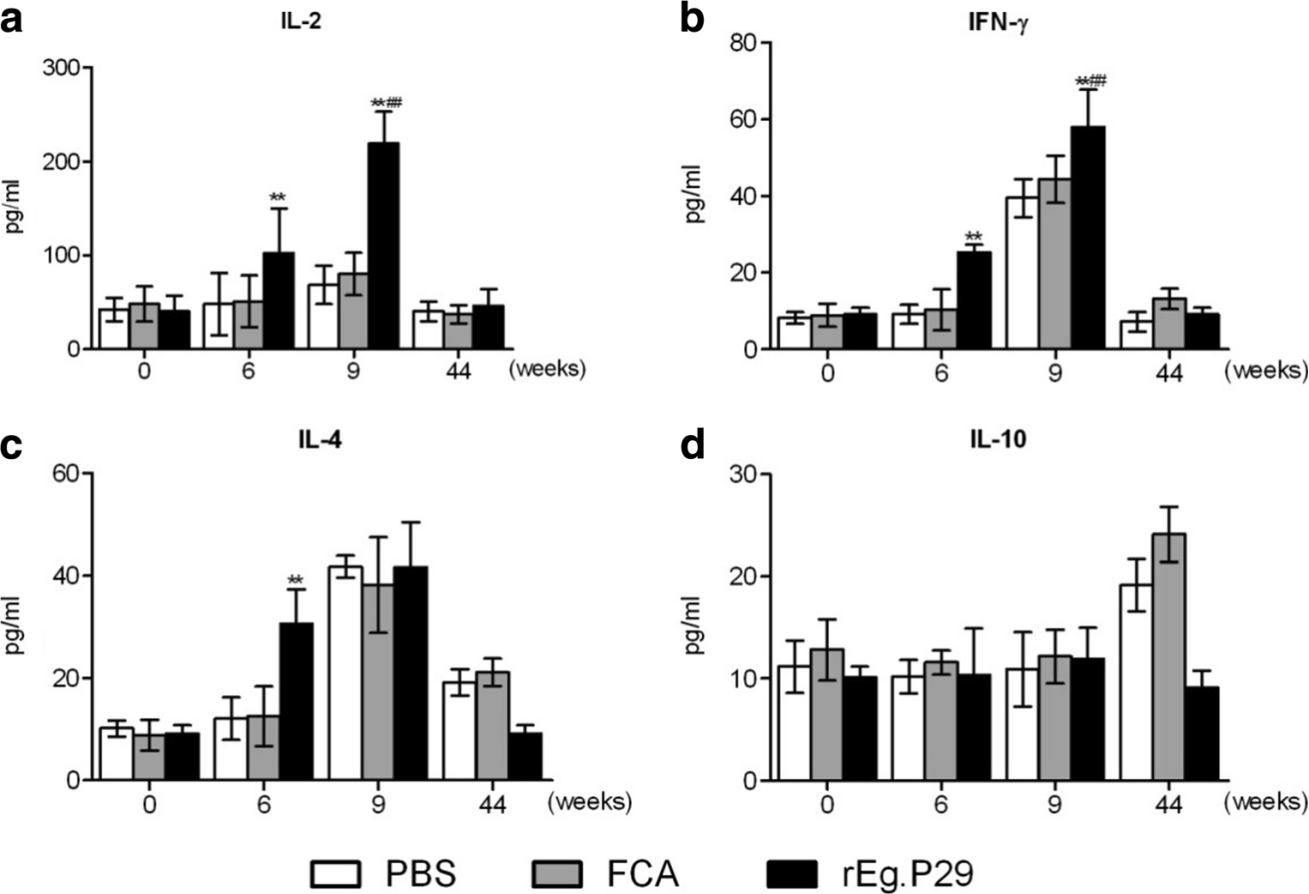
Detection of cytokines in immunized sheep before and after the infection. Different stages were selected for cytokine examination: week 0 (before immunization), week 6 (2 weeks after the second immunization), week 9 (1 week after infection), and week 44 (36 weeks after infection). *P < 0.05 and **P < 0.01 indicate significant differences between PBS and r*Eg*.P29 groups. ^#^ *P* < 0.05 and ^##^ *P* < 0.01 denote significant differences between FAC and r*Eg*.P29 groups. The assay for cytokine analysis was repeated five times.

IFN-γ, IL-4, and IL-10 levels in serum as indicators of Th1 and Th2 immune polarization were measured; IL-2 was used as a marker of T lymphocyte proliferation (Dematteis et al. 1999). IL-2 and IFN-γ levels in the r*Eg*.P29 group increased statistically compared with that in PBS or FCA groups (*P* < 0.01) at week 6 (2 weeks after the boost immunization) and then sequentially increased at week 9 (1 week after the infection) (Fig. 4a and b). Serum IL-4 levels in vaccinated sheep were higher than those in the PBS group after the immunization (*P* < 0.01) and peaked after the infection(*P* < 0.01). IL-10 level did not significantly increase in the immunized group compared with that in the control group (Fig. 4d) (*P* > 0.05).

## Discussion

The present study explored the protective effects of immunizating sheep with r*Eg*.P29 against the challenge with *E. granulosus* eggs, and the associated immune response. It’s not the first time to confirm a vaccine in sheep. Before the study, vaccination of sheep and other livestock with EG95 has been proven to generate up to 95 % protective efficacy (Lightowlers et al. 1999; Heath et al. 2012b). Furthermore, the immunological mechanism of vaccine EG95 has been investigated widely. In our study, evidently, immunization with r*Eg*.P29 leads to effective immunoprotection compared with the other two control groups. That will provide an experimental foundation for r*Eg*.P29 to be a potential vaccine.

Specific IgG, IgG1, IgG2, and IgE levels significantly increased in sheep immunized with r*Eg*.P29. This finding indicated that prime-boost immunization with r*Eg*.P29 induced potent IgG-predominant immune responses against *E. granulosus* infection. This result is consistent with EG95 vaccination effects in sheep (Heath et al. 2003; Heath and Koolaard 2012). Protection of EG95-vaccination sheep against challenge infection with *E. granulosus* were IgG-derived and complement-dependent (Gauci et al. 2005). But in the present study, whether generated IgG activates complement system for lysis of the parasite, need further studies. Furthermore, studies have reported that determination of anti-P29 IgG levels of patients with CE in post-surgical follow-up could be a valuable prognostic tool for clinical management of human CE cases (Boubaker et al. 2014). Elevated IgE in this study may stimulate mast cells and basophils for elimination of the parasite as previously reported (Pirestani et al. 2014). No significant difference of serum IgM (data not shown) indicates that r*Eg*.P29 cannot induce this antibody generation.

Cytokine response indicates Th1/Th2 polarization, which plays a crucial role in cystic localization and clinical stage (Zhang et al. 2008; Zhang et al. 2012). General speaking, Th1 cytokines induce protective cellular immune response, and Th2 cytokines promote the stimulation of humoral immune response that are responsible for parasite evasion from immune surveillance (Brunetti and Junghanss 2009; McManus et al. 2012). In our study, both IFN-γ and IL-4 levels evidently increased after the vaccination, indicating that both Th1 and Th2 immune response have been activated by r*Eg*.P29. Significant increase of IFN-γ at 9th week (one week after infection) can be as an indicator of greater Th1 than Th2 response. Significant elevated level of IL-4 at 6th week (two weeks after vaccination) suggests that IL-4 is mainly induced by rEg.P29. IL-4 level at 9th week (one week after infection) in vaccination group showed no significant difference with other two control groups, one possible explanation is either early infection induced Th1-polarized immune response that inhibit the Th2 levels or infection induced strong Th2 immune response in the control group, which covers up the slow increase of IL-4 levels in the vaccinated group. Studies manifested that Th1 dominated at the early stage of the infection and subsequently Th2 is predominant in the late of the infection (Zhang et al. 2008). In our studies, no significant difference of IL-10 between the before- and after- immunization was found, compared with the two control groups. Earlier researches on effects of IL-10 in *E. granulosus* infection or other recombinant vaccine candidates’ tests support our result (Rigano et al. 2007; Fraize et al. 2005; Ortona et al. 2003). We found high level of IL-10 at the end of chronic infection, and this may be involved with evasion of *E. granulosus* to host immune response as previous reported (Amri et al. 2009).

In the present study, there was no significant difference between FCA group and PBS group in cyst reduction, antibody and cytokine response, demonstrating that this immunoadjuvant could not induce immune response against the parasite infection directly. How the adjuvant is participating in immunoprotection of r*Eg*.P29 is unclear. Because this adjuvant is not commonly used in sheep immunization, we will optimize our immunization strategy in the further studies by selecting effective Quil A as adjuvant according to mature method in EG95 associated references (Heath et al. 2003; Heath and Koolaard 2012; Lightowlers and Heath 2004).

In summary, immunization with r*Eg*.P29 induced protective immunity against challenge of sheep with natural infection of *E.granulosus* eggs. This immunoprotection is due to humoral and cellular immune responses. The vaccine may prevent sheep transmission of the parasite and impede the natural parasite life cycle.
